# Differentiating pancreatic ductal adenocarcinoma and autoimmune pancreatitis using a machine learning model based on ultrasound clinical features

**DOI:** 10.3389/fonc.2025.1505376

**Published:** 2025-02-19

**Authors:** Lihua Zhang, Xiang Chen, Zhikui Chen, Weiji Chen, Jianmei Zheng, Minling Zhuo, Xing Chen

**Affiliations:** ^1^ Shengli Clinical Medical College of Fujian Medical University, Fuzhou, Fujian, China; ^2^ Department of Ultrasound, Fujian Provincial Hospital, Fuzhou, Fujian, China; ^3^ Department of Ultrasound, Fuzhou University Affiliated Provincial Hospital, Fuzhou, Fujian, China; ^4^ Department of Ultrasound, Fujian Medical University Affiliated Union Hospital, Fuzhou, Fujian, China; ^5^ Department of Geriatrics, First Affiliated Hospital of Fujian Medical University, Fuzhou, Fujian, China; ^6^ Department of General Surgery, Fujian Provincial Hospital, Fuzhou, Fujian, China; ^7^ Department of General Surgery, Fuzhou University Affiliated Provincial Hospital, Fuzhou, Fujian, China

**Keywords:** pancreatic ductal adenocarcinoma, autoimmune pancreatitis, ultrasound clinical features, reader study, random forest

## Abstract

**Purpose:**

This study aimed to construct a differential diagnostic model to distinguish autoimmune pancreatitis (AIP) from pancreatic ductal adenocarcinoma (PDCA) using ultrasound clinical features and machine learning algorithms.

**Methods:**

Retrospective ultrasound clinical data of patients with AIP and PDCA from three different centers were used as the training cohort, external validation cohort 1, and external validation cohort 2. Feature selection was conducted via variance filtering and LASSO regression, followed by the construction of a random forest (RF) model. The hyperparameters were optimized in the training cohort, and the final model was evaluated in the external validation cohorts. The model’s performance was assessed using sensitivity, specificity, positive predictive value (PPV), negative predictive value (NPV), the F1 score, accuracy, and area under curve(AUC). The clinical application value of the model was clarified through a comparison between humans and machines.

**Results:**

An RF model was constructed using six features: Ca 19-9 level, abdominal pain, jaundice, focal/diffuse-type AIP, blood flow signals, and morphology. In external validation cohort 1, the model’s sensitivity, specificity, PPV, NPV, F1 score, accuracy, and AUC were 86.0%, 80.0%, 81.0%, 86.0%, 84.0%, 83.0%, and 89.0%, respectively; in external validation cohort 2, these values were 72.0%, 94.0%, 93.0%, 77.0%, 81.0%, 83.0%, and 91.0%, respectively. The predictive performance of experienced radiologists using clinical information and ultrasound images demonstrated a sensitivity of 81%, specificity of 79%, PPV of 78%, NPV of 76%, F1 score of 80%, and accuracy of 80%. For radiologists with intermediate experience, the sensitivity was 75%, specificity was 74%, PPV was 73%, NPV was 76%, F1 score was 74%, and accuracy was 75%. less experienced radiologist had a sensitivity of 55%, specificity of 56%, PPV of 62%, NPV of 49%, F1 score of 58%, and accuracy of 55%.

**Conclusion:**

The RF model constructed using clinical ultrasound features achieved diagnostic levels comparable to those of experienced radiologists and can assist in differentiating AIP from PDCA, potentially guiding clinical practice.

## 1 Introduction

Pancreatic ductal adenocarcinoma (PDCA) is the most common pathological type of pancreatic cancer, accounting for 80–90% of all pancreatic cancers. PDCA has become the third-leading cause of cancer-related deaths in the United States, surpassing breast cancer (1). Chemotherapy and surgery are the main treatment options for PDCA. AIP is a rare type of pancreatitis characterized by distinctive histological features and a positive response to steroids. In 1995, Yoshida and colleagues first described autoimmune pancreatitis (AIP) (2). Due to the elevated IgG4 levels and associated systemic diseases often observed in patients with AIP, AIP is classified as an IgG4-related disease (IgG4-RD) (3). In 2011, the International Association of Pancreatology released the International Consensus Diagnostic Criteria (ICDC) for AIP. After existing standards were reviewed, criteria based on imaging of the pancreatic parenchyma and ducts, serology, involvement of other organs, pancreatic histopathology, and response to corticosteroid treatment were established (4). AIP is a type of autoimmune-mediated pancreatitis characterized by pancreatic enlargement, obstructive jaundice, lymphoplasmacytic infiltration combined with fibrosis, and a significant response to steroid treatment. The main symptoms of AIP are obstructive jaundice and abdominal discomfort. Most clinical symptoms and imaging findings lack distinctive specificity, especially in mass-forming AIP, which is often misdiagnosed as pancreatic cancer. This misdiagnosis leads to the unnecessary use of medical resources and surgical procedures.

Ultrasound examination, as a convenient, real-time, and cost-effective diagnostic tool, holds significant value for the diagnosis of AIP. The typical ultrasound manifestations of AIP include diffuse, focal, and multifocal types. The characteristic appearance of diffuse AIP on two-dimensional ultrasound is sausage-like pancreatic enlargement with reduced echogenicity, with or without main pancreatic duct dilation. However, the two-dimensional ultrasound appearance of mass-forming AIP often overlaps with that of pancreatic cancer, presenting as single or multiple hypoechoic masses, which frequently leads to misdiagnosis. Experienced ultrasound radiologists integrate patient history and clinical manifestations, carefully observe sonographic features, and consider indirect signs such as the degree of bile duct and main pancreatic duct dilation, involvement of surrounding organs, and the presence of retroperitoneal lymph node enlargement, to comprehensively differentiate these two diseases. An ultrasound diagnosis is dependent on the operator’s experience. Due to the rarity of AIP, primary ultrasound radiologists may have limited awareness and diagnostic experience regarding this disease. The serum IgG4 levels should be measured in patients with suspected AIP. Serum IgG4 elevation is the most specific serological indicator for the diagnosis of IgG4-AIP. The clinical incidence of PDCA is much higher than that of AIP; therefore, routine serum IgG4 testing for all patients is economically unfeasible. Additionally, while serum IgG4 levels are a specific indicator for diagnosing AIP, they are not elevated in all patients with AIP, and elevated levels may be observed in patients with other conditions. Therefore, IgG4 levels alone cannot provide a definitive diagnosis (5).

**Figure 1:**
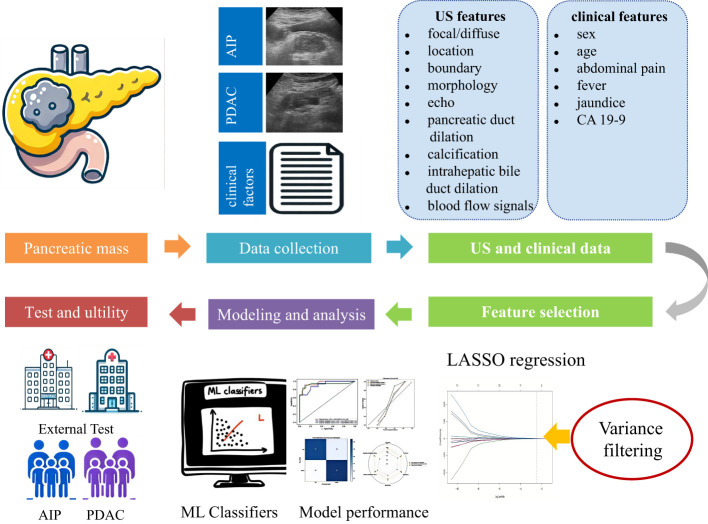
The workflow is shown.

Machine learning has been widely applied across various fields, including computer science, statistics, and information theory. It uses algorithms to analyze data, explore hidden patterns, and predict new information with high accuracy, speed, and scalability. These attributes have shown significant potential in medical diagnosis, natural language processing, and image recognition. Machine learning technology reduces the dependence on operators, standardizes image interpretation, provides stable results, enables quick decision-making, and alleviates the heavy workload of radiologists. Interest in applying machine learning technology to ultrasound has grown exponentially in recent years. This study aims to (1) explore the diagnostic value of clinical ultrasound features for the differentiation of PDCA and AIP, (2) develop a quantitative machine learning model using clinical ultrasound features to differentiate PDCA and AIP, and (3) clarify the clinical auxiliary diagnostic value via a comparison between humans and machines. The workflow is shown in(**Figure 1**).

## 2 Materials and methods

### 2.1 Patients

**Figure 2:**
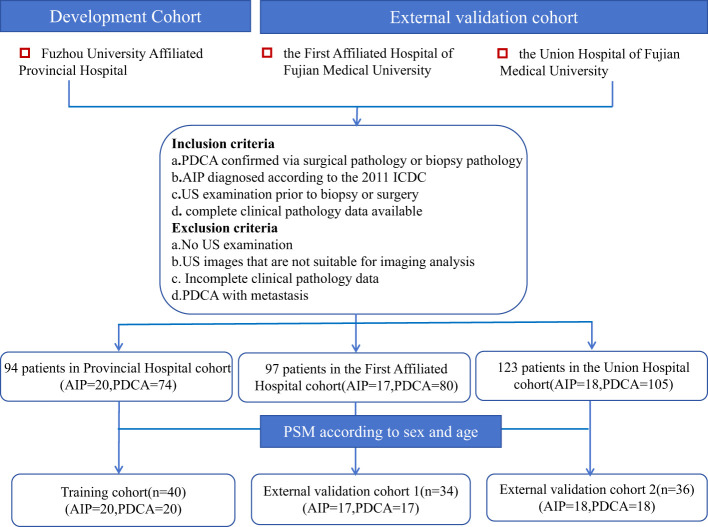
A flowchart of the patient selection process.

This multicenter retrospective study was approved by the ethics committees of Fuzhou University Affiliated Provincial Hospital, the First Affiliated Hospital of Fujian Medical University, and the Union Hospital of Fujian Medical University, and the requirement of informed consent was waived. We systematically reviewed the computer databases regarding AIP at the three hospitals from July 2014 to September 2024, identifying 90, 70, and 85 patients with AIP, respectively. We also reviewed the computer databases for patients with PDCA confirmed via surgery or biopsy from March 2022 to June 2024 at these hospitals; these patients were used as controls. We identified 100, 105, and 125 patients with PDCA from the three hospitals. The data were screened according to the inclusion and exclusion criteria. Inclusion criteria: (1) PDCA confirmed via surgical pathology or biopsy, or AIP diagnosed according to the 2011 ICDC; (2) ultrasound examination prior to biopsy or surgery; and (3) complete clinical pathology data available. Exclusion criteria: (1) no ultrasound examination prior to biopsy or surgery; (2) ultrasound images not suitable for imaging analysis; (3) incomplete clinical pathology data; or (4) PDCA with distant metastasis. Finally, 20, 17, and 18 patients with AIP and 74, 80, and 105 patients with PDCA were included from the hospitals, respectively. The clinical records, ultrasound images, and pathological data were recorded. AIP has been reported to occur mostly in middle-aged and elderly men (6). Propensity Score Matching(PSM) was used to match the patients with AIP and PDCA at a ratio of 1:1 according to sex and age, resulting in 40, 34, and 36 matched pairs from the three hospitals. The cohort from Fuzhou University Affiliated Provincial Hospital was used as the training cohort, the cohort from the First Affiliated Hospital of Fujian Medical University was used as external validation cohort 1, and the cohort from the Union Hospital of Fujian Medical University was used as external validation cohort 2. A flowchart of the patient selection process is provided (**Figure 2**).

### 2.2 Ultrasound image acquisition and analysis

Ultrasound examinations were performed using Philips IU22, Philips EPIQ5, SIEMENS S3000, and Mindray DC-8 color Doppler ultrasound diagnostic instruments and an abdominal convex array probe with a frequency of 1.0–6.0 MHz. Patients fasted for at least eight hours prior to the examination. During the examination, patients were placed in a supine position, and the convex array probe was applied to the upper abdomen to perform two-dimensional and color Doppler ultrasound scans of the pancreas. The extent of lesion involvement, location, boundary, echo, blood flow signal grading (0, I, II, or III), and associated findings such as calcification, main pancreatic duct dilation, and intrahepatic and extrahepatic bile duct dilation were observed. Blood flow was graded according to the Alder blood flow classification (7): grade 0: no blood flow within the mass; grade I: 1–2 punctate or short rod-like blood flows within the mass; grade II: 3–4 punctate blood flows or 1 long strip-like blood flow within the mass; and grade III: 5 or more punctate blood flows or 2 or more long strip-like blood flows within the mass. Ultrasound examinations and image analyses were completed by three experienced physicians from the three centers, and the results were recorded. Representative ultrasound images are shown in (**Supplementary Material**). All ultrasound images were outputted through the hospital’s picture archiving and communication system (PACS) in DICOM format. The three ultrasound physicians re-evaluated the images and recorded the ultrasound features and clinical characteristics of each patient with AIP or PDCA using the electronic medical record platform.

### 2.3 Feature processing and selection

In this study, the CA 19-9 level is the only continuous ultrasound feature, the remaining features are categorical features. The categorical variables included lesion involvement (focal AIP or diffuse AIP), location (head, neck, body, or tail of the pancreas), number, boundary (clear or unclear), echo (hypoechoic, isoechoic, or hyperechoic), presence of calcification, main pancreatic duct dilation, intrahepatic bile duct dilation, abdominal pain, jaundice, fever, and blood flow signal grading (0, I, II, or III). Most patients had a blood flow grade of 0 or I, with very few patients having grade II blood flow, and no patients having grade III blood flow. Therefore, grade 0 was categorized separately from grade I and grade II blood flow. CA 19-9 level reported by Chang et al. (2014) (8), who suggested that a CA 19-9 value > 85 U/ml had the best accuracy (85.6%) in differentiating pancreatic cancer from AIP, were used in this study. We used 85 U/ml as the cut-off value to divide the patients into two groups. During the screening process, the variance filtering method was first used to exclude variables with a variance threshold of 0. LASSO regression feature screening was then used to eliminate collinear variables.

### 2.4 Model construction and performance evaluation

During the model training phase, 10-fold cross-validation and grid search were used to optimize the hyperparameters in the training set from Fuzhou University Affiliated Provincial Hospital. The highest-scoring hyperparameters were selected as the model parameters. The constructed model was independently evaluated using datasets from the First Affiliated Hospital of Fujian Medical University and Fujian Union Hospital. The model’s performance was evaluated by calculating the sensitivity, specificity, positive predictive value, negative predictive value, F1 score, accuracy, and AUC.

### 2.5 Reader study

Three ultrasound radiologists with 10, 5, and 2 years of experience differentiated between AIP and PDCA using the ultrasound images and clinical information. The ultrasound radiologists were blinded to the pathological results. The sensitivity, specificity, positive predictive value, negative predictive value, F1 score, and accuracy of the ultrasound radiologists’ diagnoses were determined. The performance of the machine learning model was compared with the predictions of the experienced radiologist (10 years), the intermediately experienced radiologist (5 years), and the less experienced radiologist (2 years).

### 2.6 Statistical analysis

Model construction was performed using Python software (version 3.9.13), and all statistical analyses were conducted using R software (version 4.3.2). Normally distributed continuous data are expressed as the mean ± standard deviation and were compared between the two groups using the Student’s t-test. Non-normally distributed continuous data are expressed as the median [upper quartile, lower quartile] and were compared between the two groups using the Wilcoxon test. Categorical data are expressed as frequency or percentage and were compared using Fisher’s exact test. ROC curves were used to compare the models, and the DeLong test was used to compare the ROC curves. A p- value <0.05 was used to indicate statistical significance.

## 3 Results

### 3.1 Patient characteristics

A total of 110 patients were included in this study, with 55 patients with AIP and 55 patients having PDCA (101 males and 9 females; age range: 36–87 years). The CA 19-9 level, presence of abdominal pain, jaundice, focal/diffuse-type AIP, and morphology were significantly different between the AIP and PDCA groups (**Table 1**). There were no significant differences in sex or age between the AIP and PDCA groups.

**Table 1:** Patient Characteristics.

Characteristics	AIP (n=55)	PC (n=55)	p-value
Age (yr ,Mean± SD)	62.0± 12.0	62.0 ± 10.1	0.993
Sex			
Male	50 (90.9)	51 (92.7)	1
Female	5 (9.1)	4 (7.3)	
Ca199(U/ml,median IQR)	18.1 (2.8-54.7)	204.0 (32.5-735.5)	<0.001
Serum IgG4 (g/L,Mean± SD)	8.8±7.7	–	–
Abdominal pain			
Positive	25 (45.5)	42 (76.4)	0.002
Negative	30 (54.5)	13 (23.6)	
Jaundice			
Positive	26 ( 47.3)	5 (9.1)	<0.001
Negative	29 (52.7)	50 (90.0)	
Focal/Diffuse-type AIP			
Focal	32 (58.2)	54 (98.2)	<0.001
Diffuse	23 (41.8)	1 (1.8)	
Location of the lesion			
Head	34 (61.8)	25 (45.5)	0.126
Body/Tail	21 (38.2)	30 (54.5)	
Boundary			
Clear	21 (38.2)	13 (23.6)	0.148
Unclear	34 (61.8)	42 (76.4)	
Morphology			
Regular	34 (61.8)	12 (21.8)	<0.001
Irregular	21 (38.2)	43 (78.2)	
Pancreatic Duct Dilatation			
Positive	17 (30.9)	19 (34.5)	0.839
Negative	38 (69.1)	36 (65.5)	
Intrahepatic Bile Duct Dilatation			
Positive	16 (29.1)	10 (18.2)	0.262
Negative	39 (70.9)	45 (81.8)	
Blood flow signals			
Grade 1/2/3	33 (60.0)	32 (58.2)	1
Grade 0	22 (40.0)	23 (41.8)	

Except where indicated, data are numbers of patients, with percentages in parentheses.

AIP, autoimmune pancreatitis; PC, pancreatic cancer; SD, standard deviation; IQR,interquartile range.

### 3.2 Predictive performance of individual ultrasound clinical features

**Figure 3:**
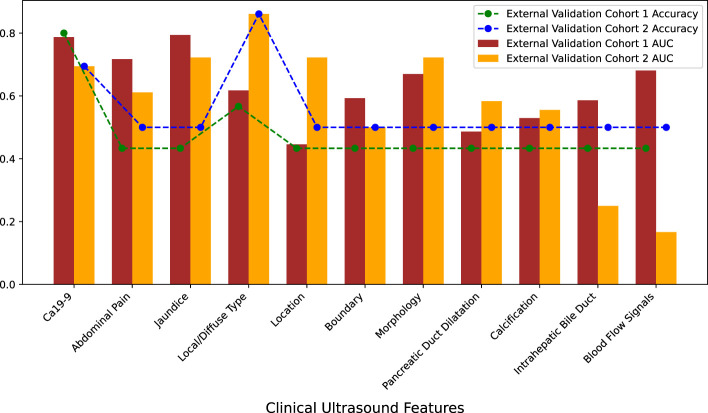
Predictive performance of individual clinical ultrasound features in external validation cohorts.

This study included 15 ultrasound clinical features: sex, age, abdominal pain, fever, jaundice, focal/diffuse type, location of the lesion, boundary, morphology, echo of the lesion, pancreatic duct dilation, calcification, intrahepatic bile duct dilation, blood flow signals, and Ca 19-9 level. PSM was used to match patients with AIP and patients with PDCA at a ratio of 1:1 based on sex and age. Almost all patients with AIP or PDCA did not have fever, and the masses were hypoechoic. Therefore, sex, age, fever, and echo were initially excluded. To understand the independent predictive performance of the ultrasound clinical features, logistic regression models were constructed separately for the remaining 11 features, and their predictive performances were evaluated using the external validation cohorts. The AUCs and accuracies of the models constructed with individual features were not high, and there was severe overfitting (**Figure 3**). The specific performance indicators of each feature are shown (**Table 2**). These findings indicate that individual ultrasound clinical features cannot be used to accurately differentiate AIP and PDCA.

**Table 2:** Single feature effectiveness analysis.

	Sensitivity, %	Specificity, %	PPV, %	NPV, %	Accuracy, %	AUC, %
External Validation cohort 1						
Sex	0	100	–	43	43	49
Age	0	100	–	43	43	37
Ca199	88	69	79	82	80	85
Abdominal pain	0	100	–	43	43	72
Jaundice	0	100	-	43	43	79
Focal/diffuse type	24	100	100	50	57	62
Location of the lesion	0	100	–	43	43	45
Boundary	0	100	–	43	43	59
Morphology	0	100	–	43	43	67
Pancreatic Duct Dilatation	0	100	-	43	43	49
Calcfication	0	100	–	43	43	53
Intrahepatic Bile Duct Dilatation	0	100	–	43	43	59
Blood flow signals	0	100	–	43	43	68
External Validation cohort 2						
Sex	0	0	–	50	50	53
Age	39	89	78	59	64	56
Ca199	72	67	68	71	69	71
Abdominal pain	0	100	–	50	50	61
Jaundice	0	100	–	50	50	72
Focal/diffuse	72	100	100	78	86	86
Location of the lesion	0	100	-	50	50	72
Boundary	0	100	–	50	50	50
Morphology	0	100	–	50	50	72
Pancreatic Duct Dilatation	0	100	–	50	50	58
Calcfication	0	100	–	50	50	56
Intrahepatic Bile Duct Dilatation	0	100	–	50	50	25
Blood flow signals	0	100	–	50	50	17

PPV, positive predictive value; NPV, negative predictive value; AUC, area under curve.

### 3.3 Feature selection and analysis

**Figure 4:**
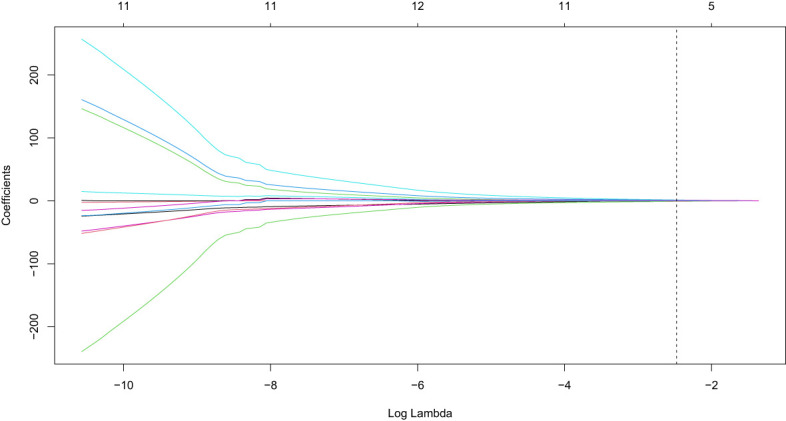
LASSO regression coefficient path for clinical ultrasound features selection.

**Figure 5:**
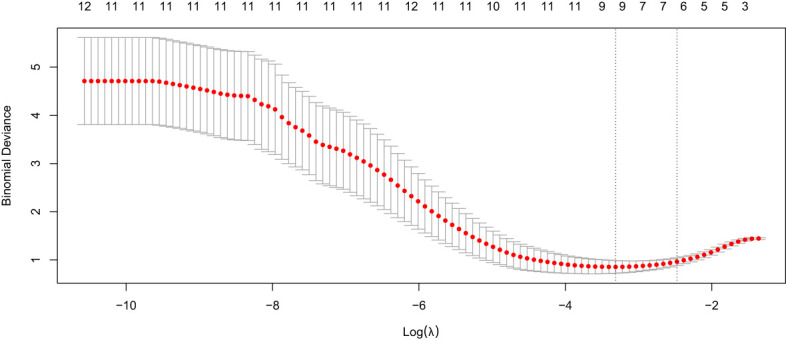
10-fold cross-validation curve for LASSO regression.

**Figure 6:**
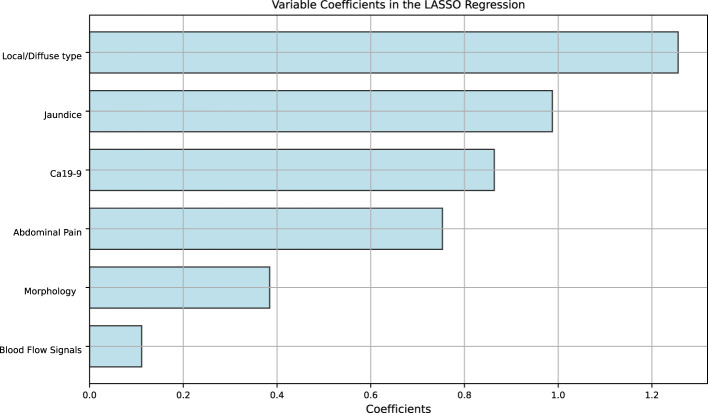
Ranking of clinical ultrasound features based on LASSO regression coefficients.

Variance filtering was used to exclude ultrasound features with a variance threshold of 0, eliminating two features: fever and echo. LASSO regression with 10-fold cross-validation was used to exclude features with a coefficient of 0, including boundary, pancreatic duct dilation, intrahepatic bile duct, location, and calcification. The remaining six features with nonzero coefficients included abdominal pain, jaundice, focal/diffuse AIP, blood flow signals, CA 19-9 level, and morphology (**Figures 4**, **5**). The six features were ranked by importance, with focal/diffuse-type AIP being the most important, followed by jaundice, CA 19-9 level, abdominal pain, morphology, and blood flow signals (**Figure 6**). (**Supplementary Material**) shows the distributions of these six features across the three datasets. Abdominal pain was more common in patients with PDCA than in those with AIP, and patients with PDCA had significantly higher CA 19-9 level than those with AIP. Diffuse lesions and jaundice were more common in the AIP group than in the PDCA group. PDCA lesions were more irregular in shape than AIP lesions. Both the PDCA and AIP groups exhibited poor blood supply, with most patients having grade 0-I blood flow signals. In the training cohort and external validation cohort 1, most patients in the AIP group had grade I blood flow signals, whereas most patients in the PDCA group had grade 0 blood flow signals. In external validation cohort 2, most patients in the PDCA group had grade I blood flow signals, whereas most patients in the AIP group had grade 0 blood flow signals.

### 3.4 Model training and validation

**Figure 7:**
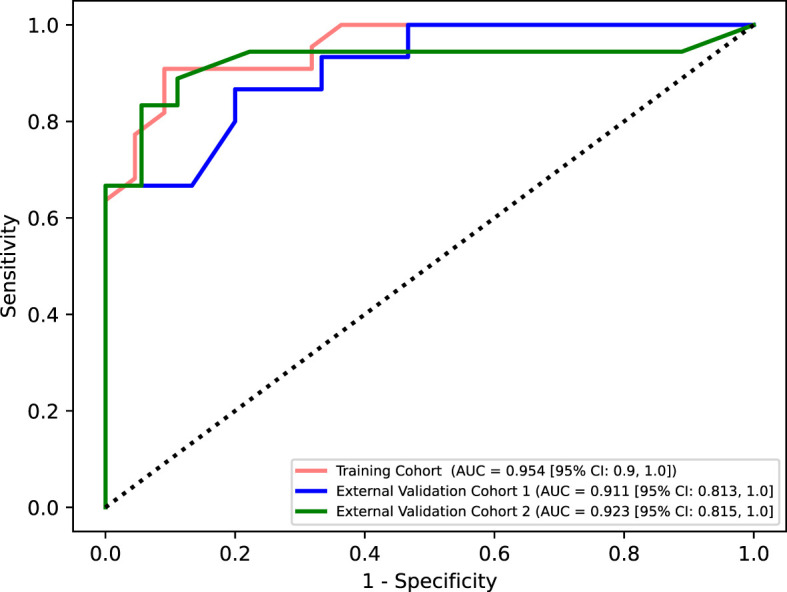
ROC curves of three cohorts.

**Figure 8:**
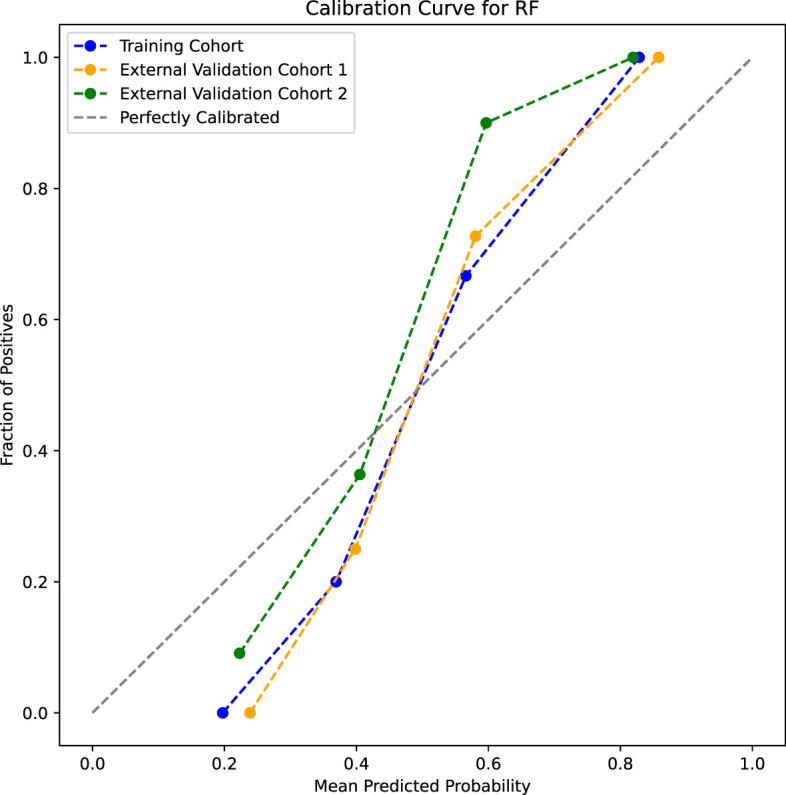
Calibration curves of the random forest model in three cohorts.

Five models were used, including the support vector machine, random forest (RF), logistic regression, decision tree, and k-nearest neighbors. Using the grid search method and 10-fold cross-validation in the sklearn library, the hyperparameters of the models were optimized in the training cohort. Among the models, RF performed the best and was selected as the final model. The parameters of the RF model were set to criterion=‘gini’, max_depth=4, min_samples_leaf=4, and n_estimators=10. The model’s sensitivity, specificity, positive predictive value, negative predictive value, F1 score, accuracy, and AUC were 86.0%, 86.0%, 86.0%, 86.0%, 70.0%, 86.0%, and 95.0%, respectively, in the training cohort. In external validation cohort 1, the sensitivity, specificity, positive predictive value, negative predictive value, F1 score, accuracy, and AUC were 86.0%, 80.0%, 81.0%, 86.0%, 84.0%, 83.0%, and 89.0%, respectively. In external validation cohort 2, the sensitivity, specificity, positive predictive value, negative predictive value, F1 score, accuracy, and AUC were 72.0%, 94.0%, 93.0%, 77.0%, 81.0%, 83.0%, and 91.0%, respectively. The DeLong test revealed no significant differences between the ROC curves of the training cohort, external validation cohort 1, and external validation cohort 2 (P>0.05) (**Table 3**, **Figure 7**). The calibration bias curves of the training cohort and external validation cohorts closely overlapped with the ideal line (**Figure 8**).

**Table 3:** Random forest model performance.

	Sensitivity,% (95% CI)	Specificity,% (95% CI)	PPV,% (95% CI)	NPV,% (95% CI)	Accuracy,% (95% CI)	AUC,% (95% CI)	F1Score,% (95% CI)
Training cohort external	86(71-100)	86(71-100)	86(71-100)	86(70–100)	86(75-95)	95(89-99)	70(74-96)
Validation cohot 1 External	86(68-100)	80(57-100)	81(62-100)	86(67-100)	83(70-97)	89(77-99)	84(68-97)
Validation Cohort 2	72(50-93)	94(82-100)	93(77-100)	77(60-94)	83(71-94)	91(79-100)	81(64-95)

PPV, positive predictive value; NPV, negative predictive value; AUC, area under curve.

### 3.5 Comparison of different models

We compared the performance differences of the model when the CA 19-9 level was included as a continuous variable or a categorical variable. The model performed better when the CA 19-9 level was included as a categorical variable with a threshold of 85 U/ml (**Table 4**). Considering the inconsistency in the distribution of blood flow signals in external validation cohort 2 compared to that of the training cohort and external validation cohort 1 and the potential influence of different equipment and operator adjustments, we attempted to exclude this feature. The model’s performance decreased when the blood flow signal feature was removed (**Table 5**).

**Table 4:** The model's performance when the CA 19-9 level was included as a continuous variable or a categorical variable with a threshold of 85 U/ml.

	Sensitivity,% (95% CI)	Specificity,% (95% CI)	PPV,% (95% CI)	NPV,% (95% CI)	Accuracy,% (95% CI)	AUC,% (95% CI)	F1Score,% (95% CI)
categorical variable	80(58-00)	80(57-100)	80(58- 100)	80(58- 100)	80(63-93)	90(78-99)	80(62-93)
continuous variable	61(38-83)	83(65-100)	79(54- 100)	68(50-87)	72(58-86)	85(71-96)	69(48-85)

PPV, positive predictive value; NPV, negative predictive value; AUC, area under curve.

**Table 5:** The model's performance when the blood flow signals feature was remained or removed.

	Sensitivity,% (95% CI)	Specificity,% (95% CI)	PPV,% (95% CI)	NPV,% (95% CI)	Accuracy,% (95% CI)	AUC,% (95% CI)	F1 Score,% (95% CI)
remained	87(68-100)	67(42-90)	72(50-92)	83(60-100)	77(60-90)	91(78-99)	79(61-91)
removed	67(44-89)	83(65-100)	80(58-100)	71(52-90)	75(61-89)	91(80-99)	73(53-88)

PPV, positive predictive value; NPV, negative predictive value; AUC, area under curve.

### 3.6 Reader study

**Figure 9:**
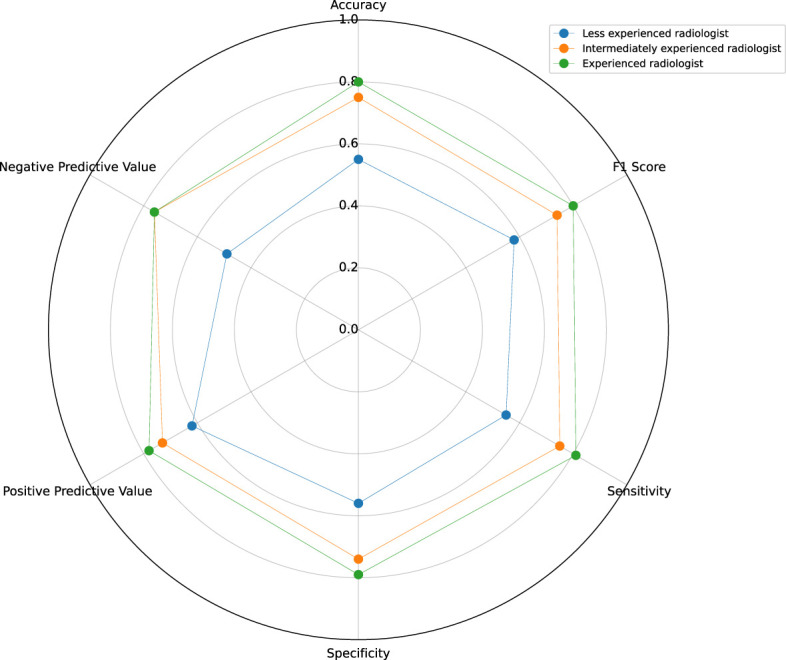
Predictive performance of radiologists with different levels of experience.

The predictive performance of experienced radiologists based on clinical information and ultrasound images showed a sensitivity of 81%, specificity of 79%, positive predictive value of 78%, negative predictive value of 76%, F1 score of 80%, and accuracy of 80%. The predictive performance of the radiologist with intermediate experience showed a sensitivity of 75%, specificity of 74%, positive predictive value of 73%, negative predictive value of 76%, F1 score of 74%, and accuracy of 75%. The predictive performance of the radiologist with two years of experience showed a sensitivity of 55%, specificity of 56%, positive predictive value of 62%, negative predictive value of 49%, F1 score of 58%, and accuracy of 55%. The classification results were visualized using a confusion matrix between the predicted and ground-truth labels (**Supplementary Material**). The experienced ultrasound radiologist’s evaluation indicators were superior to those of the radiologist with intermediate experience, and those of the ultrasound radiologist with intermediate experience were superior to those of the less experienced ultrasound radiologist (**Figure 9**).

## 4 Discussion

This study included data from three medical centers and found that clinical ultrasound features have certain value in differentiating AIP and PDCA, though individual features exhibit poor performance and overfitting issues. Through variable selection, six ultrasound features were identified: focal/diffuse-type AIP, jaundice, CA 19-9 level, abdominal pain, morphology, and blood flow signals. The RF model constructed with these features can be used for the differential diagnosis of AIP and PDCA, as AUC values of 89% and 91% were obtained in the external validation cohorts 1 and 2, respectively. The model reached the classification level of experienced ultrasound radiologists, outperforming those with intermediate and low experience. Therefore, the model can be used in primary medical institutions to assist primary care doctors in differentiating AIP and PDCA and may help avoid unnecessary biopsies and surgeries. To our knowledge, this work describes the first machine learning model based on clinical ultrasound features to differentiate AIP and PDCA, and it achieves good performance in multicenter cohorts.

Standard imaging techniques for diagnosing AIP include CT, MRI, and EUS. The typical CT manifestation of AIP includes clear, low-density, cystic margins around the pancreas, and enhanced CT typically reveals diffuse pancreatic enlargement. The typical enhanced CT manifestation of pancreatic cancer is a hypovascular focal mass. However, simple pancreatic enlargement cannot accurately differentiate AIP from PDCA and other malignancies (9). MRI is necessary to visualize the pancreatic duct and its secondary branches, with typical MRCP manifestations include multiple segmental strictures of the pancreatic duct often related to pancreatic lesions. However, MRI examination is not specific for diffuse, focal, or segmental enlargement and is more costly and technically demanding compared to CT and EUS (10). EUS combines the advantages of electronic endoscopy and ultrasound, allowing for the visualization of structures not observed via endoscopy, such as hemodynamic changes, the depth of lesion tissue infiltration, and extra-luminal organs. EUS is often used to assess the staging of gastrointestinal tumors (11). Moreover, EUS-guided fine-needle aspiration (EUS-FNA) can be used to reveal pancreatic lesion pathology and has significant value in differentiating pancreatic cancer from AIP (12). However, EUS-FNA may yield insufficient tissue samples due to the small sample size, leading to false-negative results. In addition, EUS-FNA has limitations, such as the inability of some patients to tolerate endoscopy, which prevents lesion tissue acquisition or causes secondary injuries (13–15).

In addition, serological tests play an important role in distinguishing AIP and PDCA. Hamano et al. (16). reported that a serum IgG4 concentration threshold of 1.35 g/L distinguishes AIP from pancreatic cancer, with diagnostic accuracy, sensitivity, and specificity all exceeding 95%; however, elevated serum IgG4 levels are also observed in other diseases, such as tumors and infections (17). Serum CA 19-9 level increase differently inin the presence of malignant tumors, benign tumors and inflammatory responses of the digestive system, especially in patients with pancreatic cancer, making it the most commonly used biomarker for pancreatic cancer screening. Serum CA 19-9 is expressed at low levels in normal pancreatic tissue, however, its expression gradually increases when pancreatic duct epithelial cells undergo malignant transformation, and the diagnostic sensitivity for pancreatic cancer is significantly greater than that of other tumor markers. In this study, patients with PDCA had significantly higher CA 19-9 level than patients with AIP, which is consistent with the findings of Chang et al. in 2014 (8).

Abdominal ultrasound, which is convenient, non-invasive, cost-effective, and repeatable, is a routine screening method for pancreatic lesions and provides a convenient and effective method for posttreatment follow-up of AIP. Almost all patients with AIP or PDCA undergo ultrasound examination prior to other tests, and the data provided by ultrasound doctors plays an important role in subsequent clinical decision-making. It is difficult to differentiate between AIP and PDCA using routine abdominal ultrasound alone. This study revealed that while individual clinical ultrasound features are significantly different between the two groups, models constructed with single features exhibited low AUCs and accuracies as well as significant overfitting issues. Therefore, the use of individual clinical ultrasound features to differentiate AIP and PDCA is insufficient. Ultrasound radiologists typically do not use individual ultrasound features for differential diagnosis. Therefore, clinical, serological, and ultrasound features were used to construct the model, and six features were identified via feature selection. These features were (ranked by importance): focal/diffuse-type AIP, jaundice, CA 19-9 level, abdominal pain, morphology, and blood flow signals.

Patients with PDCA had focal lesions, high CA19-9 levels, abdominal pain, irregular shapes, and grade 0 blood flow more often than those with AIP and had significant jaundice less frequently than patients with AIP. AIP often presents with painless obstructive jaundice, which may be due to external compression of the pancreatic head by inflammatory processes, leading to stenosis of the bile duct in the pancreatic region or IgG4-RD with concurrent sclerosing cholangitis (18). An international multicenter survey (19) on AIP found that the incidence of obstructive jaundice in patients with AIP is 75%. The first symptom of PDCA is often abdominal pain, and with improved awareness and instrument resolution, tumors are often detected when they are smaller, making obstructive jaundice less common in patients with PDCA than in those with AIP. According to the extent of involvement, AIP can be classified into diffuse, focal, and segmental types on ultrasound. Diffuse- type AIP is the most common characterized by significant lymphoplasmacytic infiltration and fibrosis in the pancreatic parenchyma, which eliminates the normal feathery structure and causes sausage-like swelling with fat gap loss (20). PDCA is characterized by infiltrative growth, presenting as an irregularly shaped mass on imaging. The blood flow signals were often grade 0 in patients with PDCA in this study, which may be due to tumor cell infiltration and destruction of internal blood vessels, thrombosis, and arteriovenous shunting. The AIP group had relatively more patients with grade I or II blood flow signals than the PDCA group, which may be due to the pathological changes of AIP, involving inflammatory lymphocyte infiltration, interstitial fibrosis, and fibrous tissue hyperplasia without significant destruction and proliferation of microvessels within the lesion. Therefore, the blood flow signals of the lesions are not significantly different from those of the surrounding normal pancreatic tissue.

We found that experienced ultrasound radiologists have strong differentiation abilities, with an accuracy of 0.80, which is superior to that of ultrasound radiologists with intermediate and low experience. Experienced ultrasound radiologists typically rely on years of experience to identify subtle differences in sonographic images and data regarding clinical manifestations and laboratory tests to make comprehensive diagnoses, achieving high classification accuracy. However, the diagnostic performance of a less experienced ultrasound radiologist (accuracy of 55%) was equivalent to that of random guessing as AIP is rare in clinical practice, and less experienced ultrasound radiologist lack awareness and experience. The ultrasound radiologist do not sufficiently combine laboratory indicators to recognize diseases, especially AIP. Less experienced ultrasound radiologist also have less mature machine adjustment capabilities, leading to deviations in the display of some ultrasound sonographic features, such as lesion blood flow signals and morphology. Therefore, differentiating between AIP and PDCA relies heavily on the ultrasound radiologist’s experience, requiring significant time and money for training. The model constructed in this study is expected to reduce the impact of subjective judgments when using ultrasound images for diagnoses, and it can be promoted in primary medical institutions to better differentiate AIP and PDCA, reducing unnecessary biopsies and surgeries.

Limitations of our study:

1. Despite collecting data from three centers over a ten-year follow-up period, the sample size remains limited, which increases the risk of overfitting in the machine learning models. Future studies should aim to include larger sample sizes and conduct multicenter validation to enhance the model’s generalizability.
2. The ultrasound features used in the model—such as focal/diffuse-type AIP, jaundice, CA 19-9 level, abdominal pain, morphology, and blood flow signals—are generally accessible in clinical settings. However, blood flow signals may vary due to differences in equipment or operator technique, potentially introducing bias into the model.
3. The model requires the interpretation of ultrasound features by a clinician, which necessitates professional expertise and may limit its widespread adoption, particularly in settings with less specialized staff. As data volume increases, AI could potentially assist in further refining the differentiation between AIP and PDCA, reducing the reliance on specialized knowledge.
4. Combining the predictive outputs of the machine learning algorithm with serological tests, such as IgG4 detection, could enhance the model’s diagnostic accuracy in distinguishing AIP from PDCA. Future prospective studies should validate this integrated approach.

## 5 Conclusion

The Random Forest (RF) model developed using clinical ultrasound features demonstrates significant clinical value in differentiating AIP from PDCA. Further validation and refinement of this model with larger and more diverse datasets will be crucial for its broader clinical application.

## Data Availability

The original contributions presented in the study are included in the article/**Supplementary Material**. Further inquiries can be directed to the corresponding author.
