# Supplementary figures and images for: Differentiating pancreatic ductal adenocarcinoma and autoimmune pancreatitis using a machine learning model based on ultrasound clinical features
Source: Front Oncol. 2025 Feb 19;15:1505376. doi: 10.3389/fonc.2025.1505376 (PMC11879800; doi:10.3389/fonc.2025.1505376)

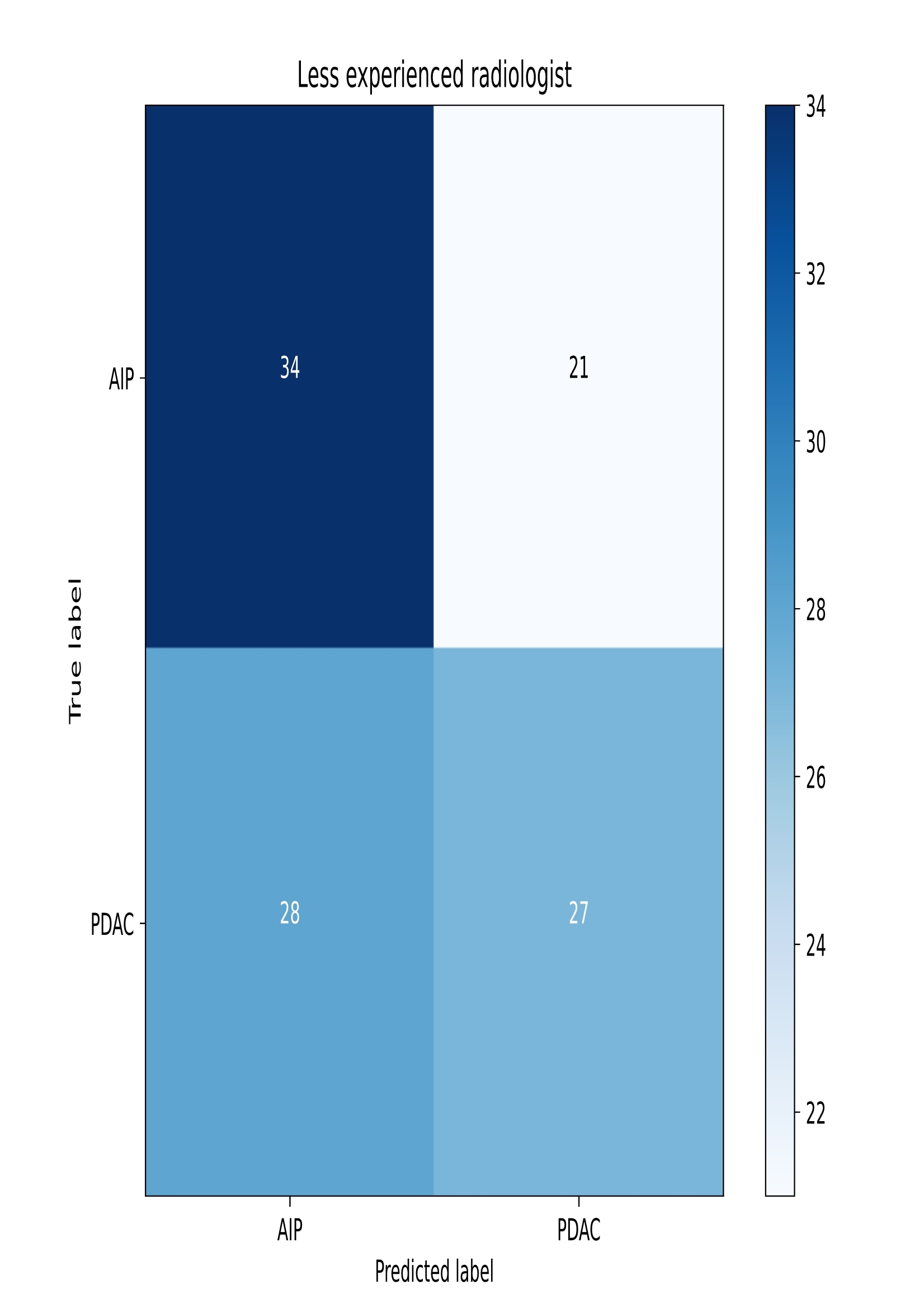

Supplement: Supplementary file 1 [file Image1.jpeg]

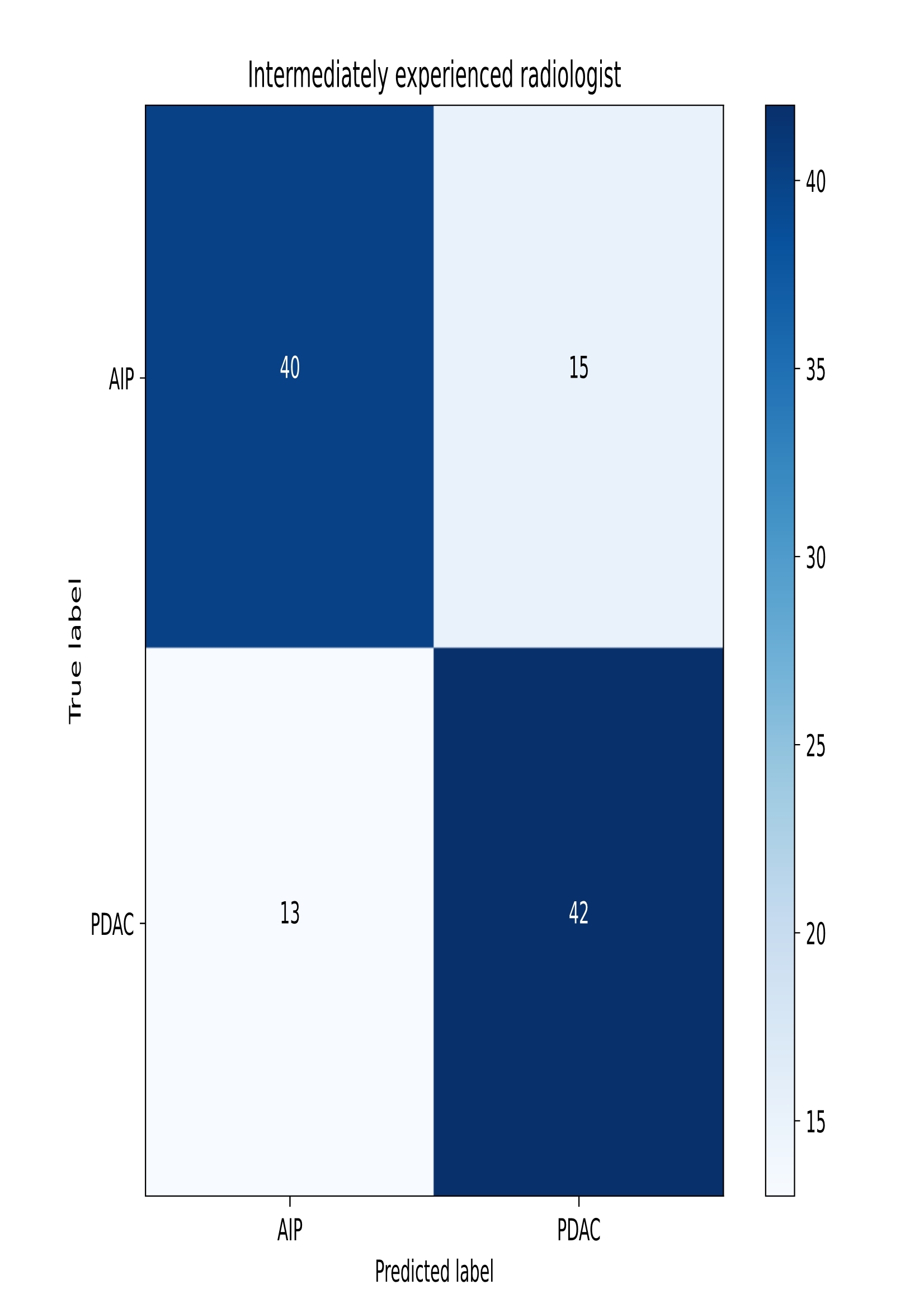

Supplement: Supplementary file 2 [file Image2.jpeg]

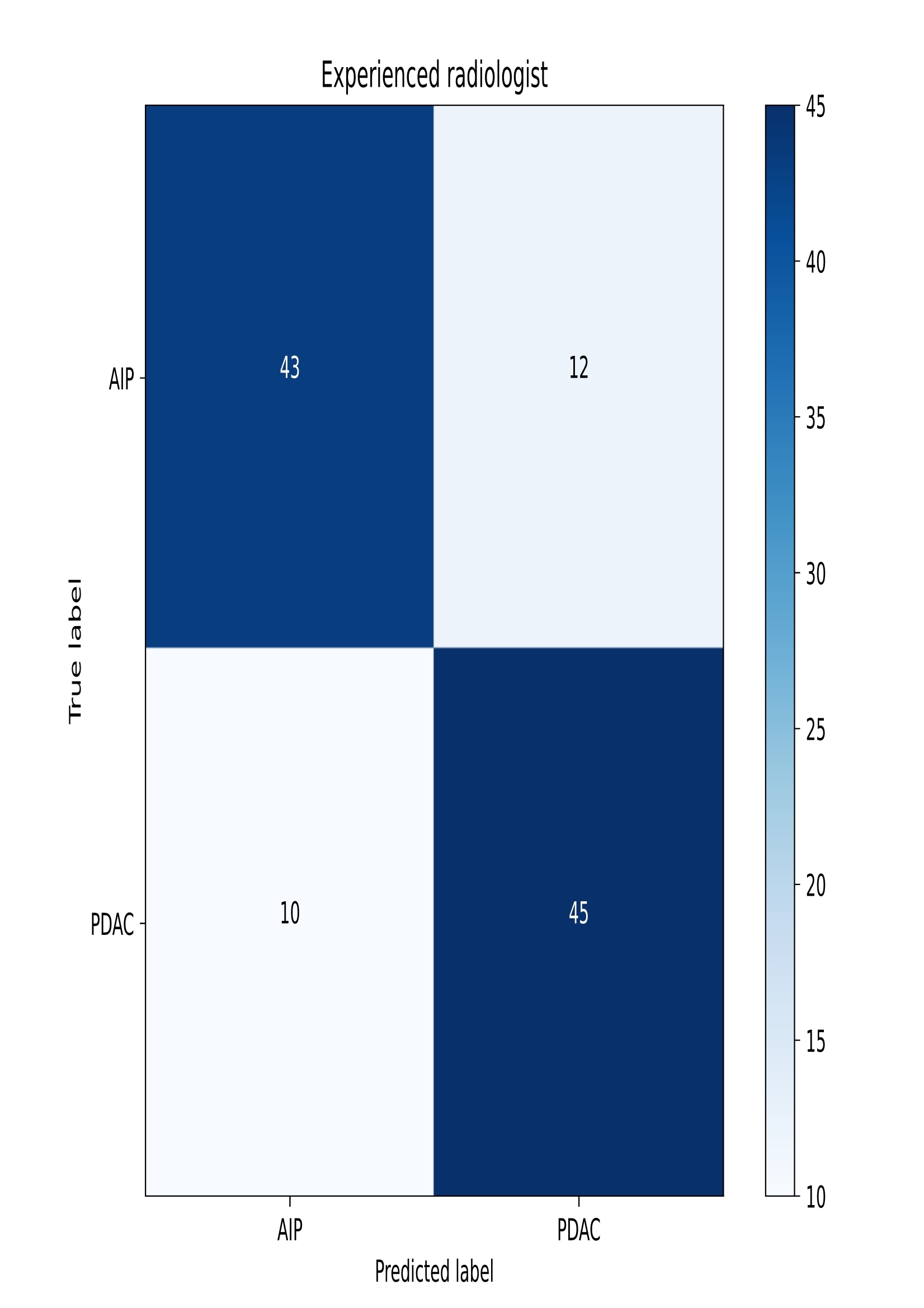

Supplement: Supplementary file 3 [file Image3.jpeg]

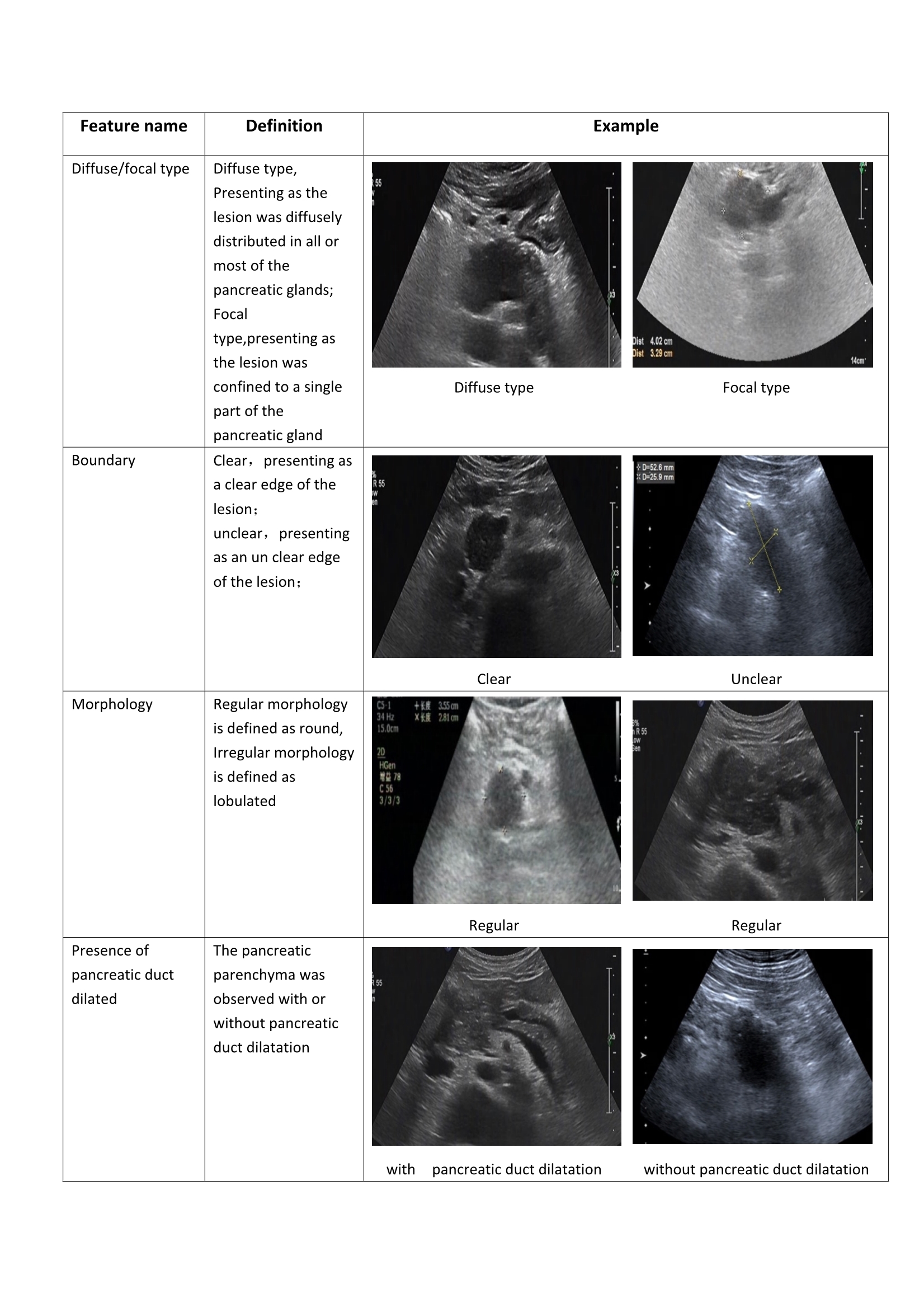

Supplement: Supplementary file 4 [file Image4.jpeg]

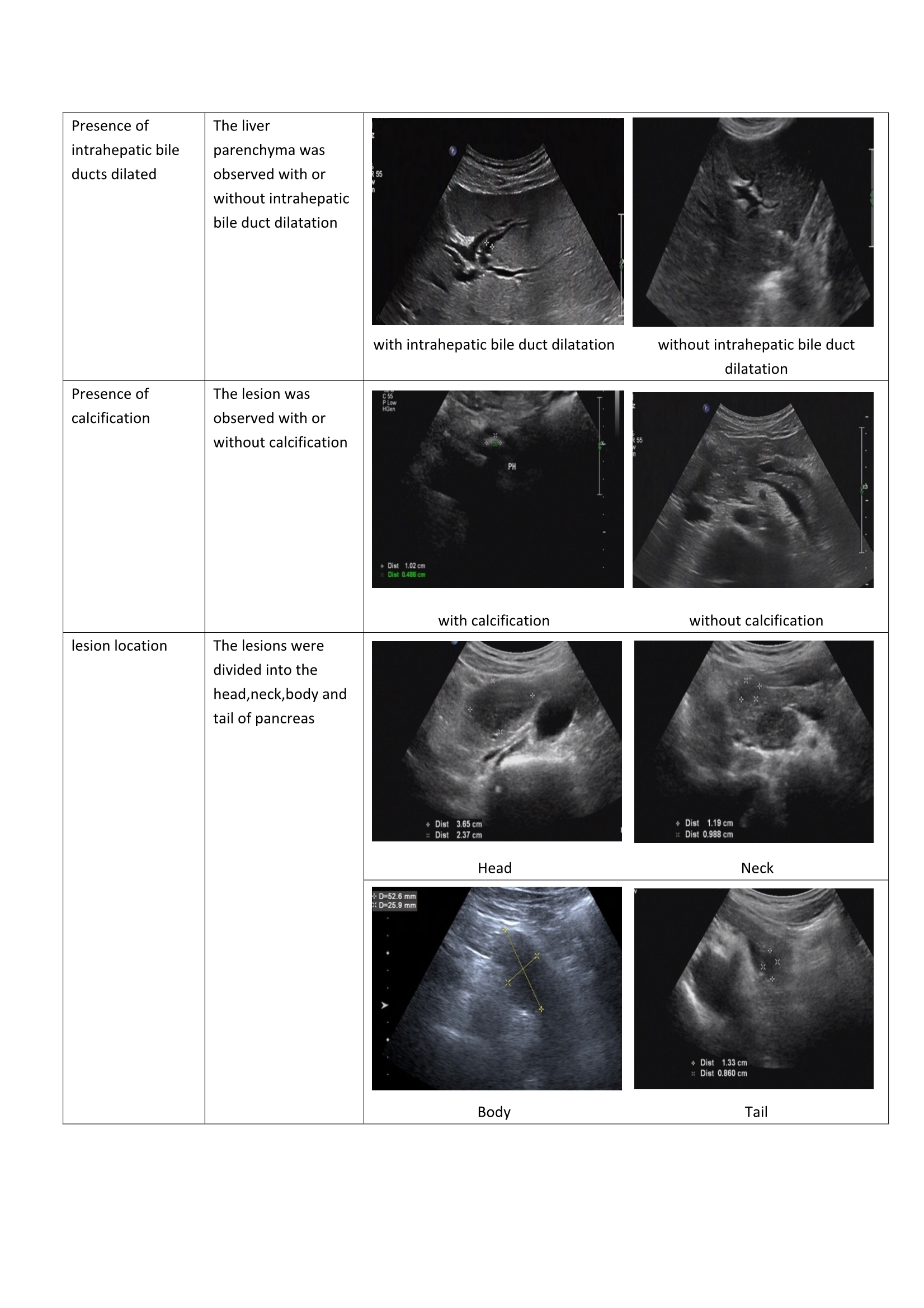

Supplement: Supplementary file 5 [file Image5.jpeg]

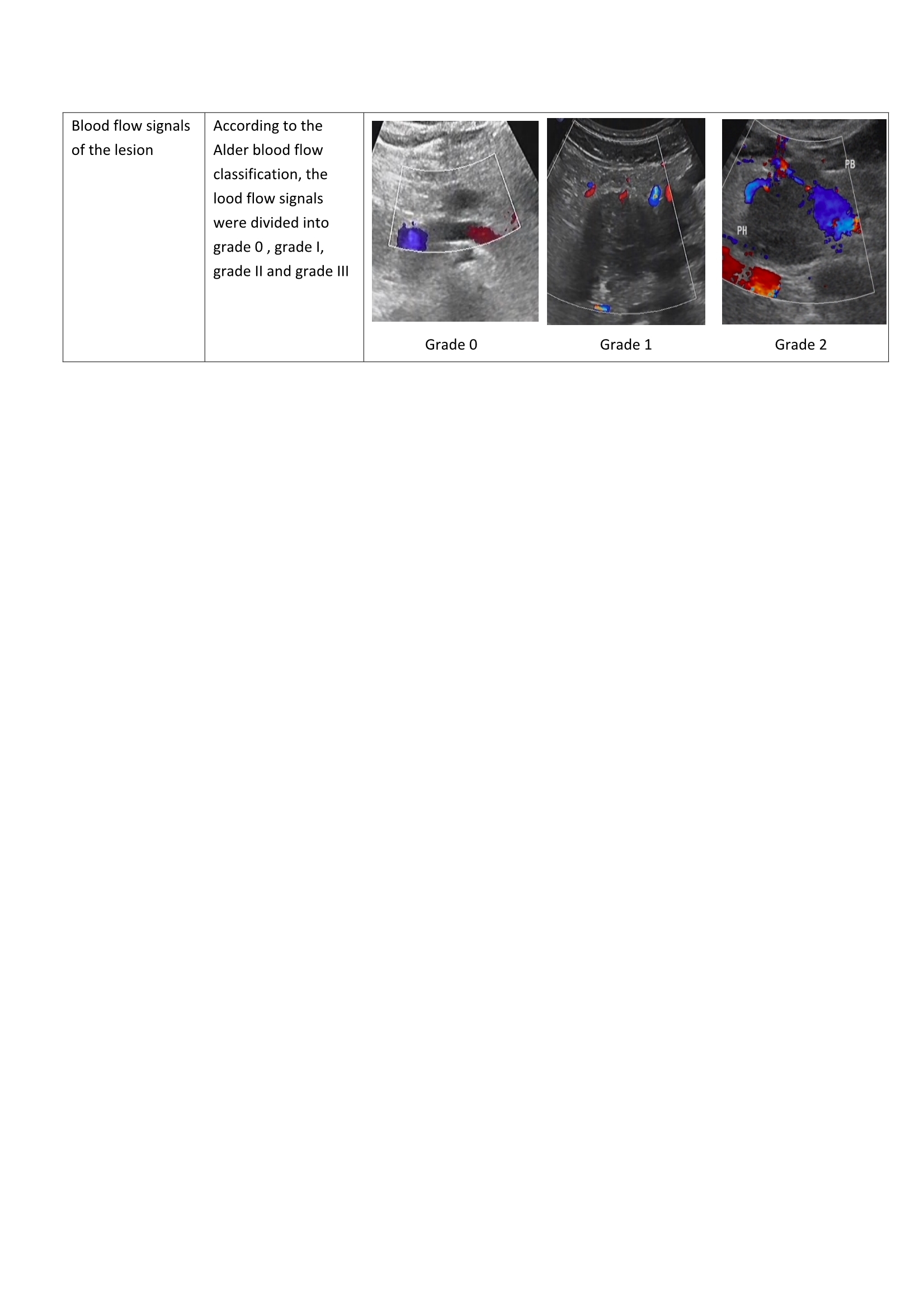

Supplement: Supplementary file 6 [file Image6.jpeg]

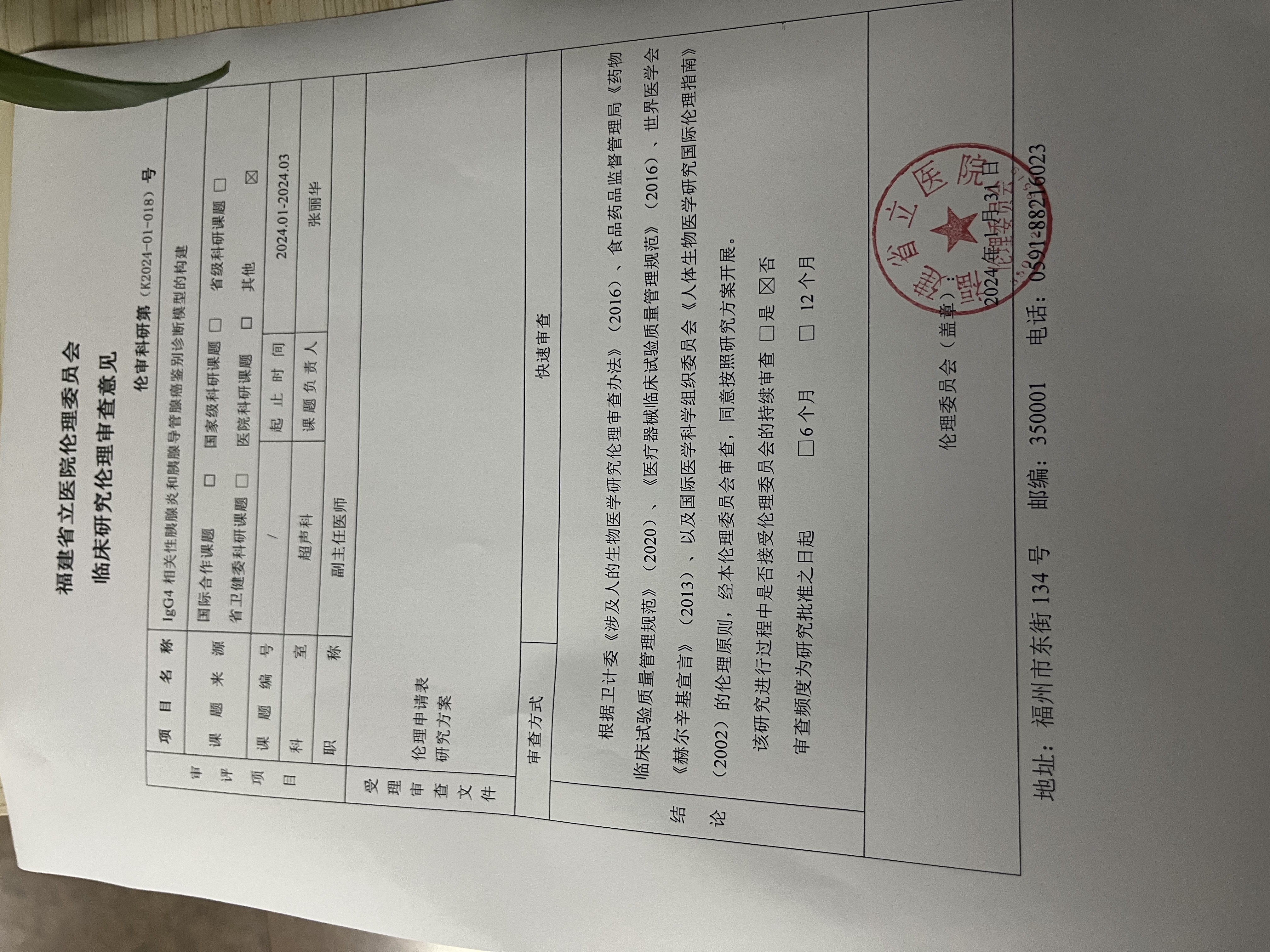

Supplement: Supplementary file 7 [file Image7.jpeg]

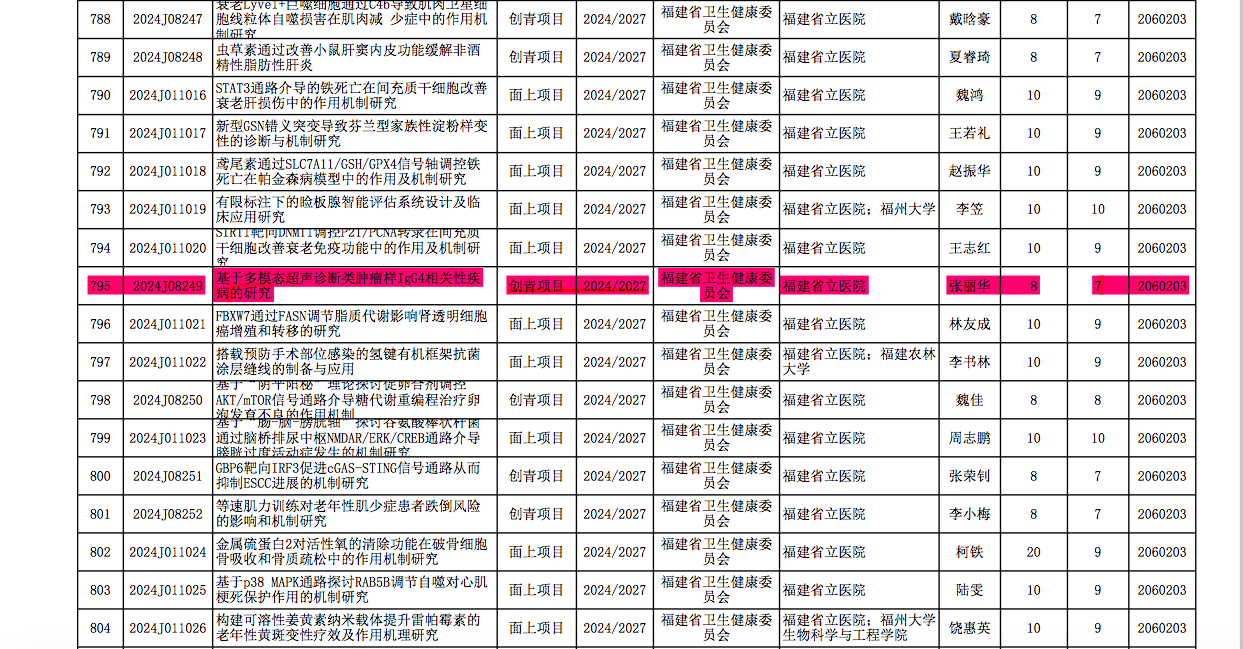

Supplement: Supplementary file 8 [file Image8.jpeg]
